# Construction and validation of a novel senescence-related risk score can help predict the prognosis and tumor microenvironment of gastric cancer patients and determine that STK40 can affect the ROS accumulation and proliferation ability of gastric cancer cells

**DOI:** 10.3389/fimmu.2023.1259231

**Published:** 2023-10-17

**Authors:** Weijie Sun, Yihang Yuan, Jiaying Chen, Qun Bao, Mengsi Shang, Peng Sun, Haixia Peng

**Affiliations:** ^1^ Digestive Endoscopy Center, Tongren Hospital, Shanghai Jiao Tong University School of Medicine, Shanghai, China; ^2^ Department of General Surgery, Tongren Hospital, Shanghai Jiao Tong University School of Medicine, Shanghai, China

**Keywords:** gastric cancer, senescence, prognostic model, immunotherapy, machine learning, Stk40, reactive oxygen species, ScRNA-seq

## Abstract

**Background:**

In recent years, significant molecules have been found in gastric cancer research. However, their precise roles in the disease’s development and progression remain unclear. Given gastric cancer’s heterogeneity, prognosis prediction is challenging. This study aims to assess patient prognosis and immune therapy efficacy using multiple key molecules.

**Method:**

The WGCNA algorithm was employed to identify modules of genes closely related to immunity. A prognostic model was established using the Lasso-Cox method to predict patients’ prognosis. Single-sample gene set enrichment analysis (ssGSEA) was conducted to quantify the relative abundance of 16 immune cell types and 13 immune functions. The relationship between risk score and TMB, MSI, immune checkpoints, and DNA repair genes was examined to predict the effectiveness of immune therapy. GO and KEGG analyses were performed to explore potential pathways and mechanisms associated with the genes of interest. Single-cell RNA sequencing was utilized to investigate the expression patterns of key genes in different cell types.

**Results:**

Through the WGCNA algorithm and Lasso-Cox algorithm selected KL, SERPINE1, and STK40 as key genes for constructing the prognostic model. The SSGSEA algorithm was employed to evaluate the infiltration of immune cells and immune functions in different patients, and their association with the risk score was investigated. The high-risk group exhibited lower TMB and MSI compared to the low-risk group. MMR and immune checkpoint analysis revealed a significant correlation between the risk score and multiple molecules. Finally, we also believe that STK40 is the most critical senescence-related gene affecting the progression of gastric cancer. *In vitro* experiments showed that ROS accumulation and cell proliferation ability of gastric cancer cells were impaired when STK40 was knocked down.

**Conclusion:**

In summary, we’ve constructed a prognostic model utilizing key genes for gastric cancer prognosis, while also showcasing its efficacy in predicting patient response to immunotherapy.

## 1 Introduction

Gastric cancer is one of the most common malignant tumors of the digestive system worldwide, ranking as the fifth most common cancer and the third leading cause of cancer-related deaths globally (1, 2). The incidence and mortality rates of gastric cancer are high, but there are significant variations among different regions (3, 4). Treatment options for gastric cancer include surgical resection, chemotherapy, radiotherapy, and targeted therapy (5). With the advancement of next-generation sequencing, treatment decisions are now influenced by tumor subtypes, overall health status of the patients, and individualized considerations (6, 7). Meanwhile, due to the heterogeneity of gastric cancer tissue, treatment outcomes can vary greatly. Therefore, new biomarkers and prognostic models are needed to facilitate precise management for individual patients.

Immunotherapy is an emerging cancer treatment modality in recent years, which harnesses various components of the immune system to combat cancer (8, 9). Major therapeutic approaches in immunotherapy include immune checkpoint inhibitors, T-cell therapy, cancer vaccines, cytokine therapy, and CAR-T cell therapy (10, 11). Immune checkpoint inhibitors, in particular, are representative treatment modalities that have demonstrated remarkable efficacy in a subset of patients. However, it is regrettable that the clinical benefits of immune checkpoint inhibitors remain limited in the majority of patients (12, 13). Immune checkpoint blockade often works by enhancing immune cell infiltration within the tumor microenvironment (14). Increasing evidence supports the critical role of immune cell infiltration in the tumor microenvironment for effective immunotherapy (15).

Senescence is a gradual process of physiological and functional changes that occur in an organism throughout its lifespan, resulting in a progressive decline in its physical and functional capabilities. Senescence is a natural biological phenomenon that affects nearly all living organisms (16). Cellular senescence is characterized by changes in gene expression and regulation, accumulation of DNA damage, decline in mitochondrial function, and increased cellular apoptosis (17, 18). Pancreatic cancer is a highly lethal and aggressive malignancy with poor prognosis. Studies have revealed the impact of CCNB1 silencing on the cell cycle, senescence, and apoptosis of pancreatic cancer cells through the p53 signaling pathway (19). Literature reports have demonstrated the close association between cellular senescence-related models and the prognosis of bladder cancer (20). Disruption of the liver microbiota balance leading to activation of hepatic stellate cells and acceleration of senescence processes contributes to the progression from liver fibrosis to hepatocellular carcinoma (21). Furthermore, the complete absence of the miR-200 family induces EMT-associated cellular senescence in gastric cancer (22). However, research on the relationship between gastric cancer and senescence is currently limited to individual molecular studies, and investigations focusing on multiple key senescence genes in gastric cancer remain limited.

In this study, we used Weighted Gene Co-expression Network Analysis (WGCNA) to screen three immune-related senescence-related genes in gastric cancer and constructed a riskscore. This risk score accurately predicted gastric cancer prognosis, and we also explored the association of this model with immune cell infiltration using bulk and single-cell RNA sequencing (scRNA-seq) analyses. Furthermore, we assessed the therapeutic effect of immunotherapy based on this risk score. Last but not least, we identified STK40 as a key senescence-related gene in gastric cancer, and determined that STK40 can affect ROS accumulation and cell proliferation in gastric cancer cells.

## 2 Materials and methods

### 2.1 Data acquisition

Transcriptomic and clinical data for gastric cancer were obtained from the TCGA database, consisting of 375 cancer samples and 32 normal samples. The clinical data included information on survival status, survival time, and other relevant variables. For further model validation and analysis, gastric cancer-related datasets (GSE84437) were downloaded from the GEO database. After data preprocessing, a total of 431 gastric cancer samples were included in the subsequent analysis.

### 2.2 WGCNA analysis

WGCNA is a computational method used to analyze gene expression patterns across multiple samples. It involves clustering genes with similar expression patterns and examining the relationships between gene modules and specific traits or phenotypes. In this study, WGCNA was utilized to construct a weighted correlation network using senescence-related genes. Specifically, modules related to the immune system were selected for subsequent analysis.

### 2.3 Modeling analysis

A risk score model was constructed using the selected key genes, and the risk score for each gastric carcinoma patient was calculated. The impact of these key molecules on the prognosis of gastric cancer patients was then evaluated. Lasso-Cox regression analysis was performed to build the prognostic model. The risk score for each patient was calculated using the formula: riskScore = [Expression of KL × coefficient] + [Expression of SERPINE1 × coefficient] + [Expression of STK40 × coefficient]. The TCGA dataset was divided into a training set and a validation set, and further model validation was conducted using the GSE84437 dataset. Kaplan-Meier (KM) curves were used for survival analysis to assess whether there were differences in survival rates between the high-risk and low-risk groups of gastric cancer patients in both the training and validation sets. Subsequently, the risk survival curves were used to evaluate the survival and mortality status of patients in the high-risk and low-risk groups, as well as the differences in the expression of key genes in the model between the two groups. ROC curves were employed to assess the performance of the predictive model. Univariate and multivariate Cox regression analyses were performed to evaluate whether different clinical indicators were valuable independent prognostic factors. Nomograph plots were used to predict the probability of gastric cancer occurrence.

### 2.4 Immune analysis and drug sensitivity analysis

The abundance of various immune cell infiltrations in each sample was quantified using the ssGSEA method. A total of 16 immune cell types and 13 immune functions were evaluated. Spearman correlation analysis was conducted to assess the relationship between the risk score model and tumor mutation burden (TMB) and microsatellite instability (MSI), indicating the model’s suitability for predicting the efficacy of immunotherapy. Mismatch Repair (MMR) and immune checkpoint analyses were used to determine the correlation between the risk score and immunotherapy. The “oncoPredict” package was utilized to evaluate the sensitivity to drugs among different groups. We assessed the relevance of chemotherapy drugs currently associated with gastric cancer.

### 2.5 Functional enrichment analysis

To explore the underlying biological processes and signaling pathways associated with differentially expressed genes, we utilized the “clusterProfiler” R package to perform gene ontology (GO) analysis, including biological processes (BP), cellular components (CC), and molecular functions (MF). Additionally, we conducted KEGG enrichment analysis using the Gene Set Enrichment Analysis (GSEA) method. The gene set file used for GO analysis annotation was “c5.go.v7.4.symbols.gmt”, and for KEGG analysis annotation, we utilized the gene set file “c2.cp.kegg.v7.4.symbols.gmt”.

### 2.6 Single-cell data analysis

The scRNA-seq data were obtained from the GEO database (GSE167297). The “Seurat” package was utilized for data analysis, including PCA dimensionality reduction and t-SNE visualization, to cluster cells based on their expression profiles. Cell types in the scRNA-seq dataset were annotated using the “SingleR” package. To investigate the intercellular communication within gastric cancer tissue, we performed cell-cell interaction analysis using the “cellchat” package. This allowed us to explore the interactions between different cell types in gastric cancer tissue.

### 2.7 Machine learning analysis

Random Forest is an ensemble algorithm that combines multiple decision trees to improve the accuracy and generalization performance of the model. It does so by using voting or averaging techniques. Both Random Forest and Lasso are well-known machine learning methods that can be used for feature gene selection. In our study, we utilized these two algorithms to identify core genes from the pool of senescence-related genes.

### 2.8 Sample collection, RNA extraction and real-time PCR reaction

10 pairs of gastric cancer tissues and adjacent normal tissues were obtained from Tongren Hospital Affiliated to Shanghai JiaoTong University and stored at -80°C for a long time. RNA extraction and quantitative PCR reaction steps are described in our previous study. The primer sequences designed in this study are detailed in **Supplementary Table 1**.

### 2.9 Cell culture and transfection

The gastric cancer cell lines HGC29 and AGS used in this study were purchased from the Cell Bank of the Chinese Academy of Sciences (Shanghai, China). All gastric cancer cells were cultured in 1640 medium containing 1% penicillin-streptomycin and 10% fetal bovine serum in a 5% CO2 incubator at 37°C. Cell transfection was performed as in our previous study. The sequence of siSTK40 is as follows, siSTK40-1: sense-CGGAUGGUUAAGAAGAUGA(dt)(dt), antisense-UCAUCUUCUUAACCAUCCG(dt)(dt). siSTK40-2: sense-GGGAGACUGUGGUAAUCUU(dt)(dt), antisense-AAGAUUACCAGUUCCCC(dt)(dt).

### 2.10 CCK8 experiment

The Cell Counting Kit-8 (CCK-8) assay is a widely used method for assessing cell viability and proliferation capabilities. It indirectly measures cell metabolic activity through a colorimetric reaction. The basic steps of the CCK-8 assay are as follows: 2000 HGC29 cells and AGS cells were planted in 96-well plates, and 10ul CCK8 reagent solution (Targetmol, USA) was added at 0h, 24h, 48h, 72h and 96h, respectively, and incubated in the dark for 2h. The absorbance was then detected at 450 nm using a microplate reader. 5 replicate holes were set up in each group (23).

### 2.11 ROS measurement

We measured the ROS activity of gastric cancer cells by detecting the fluorescence intensity of the fluorescent probe DCFH-DA (Beyotime, Shanghai, China) by flow cytometry. Briefly, gastric cancer cells were first collected in EP tubes, washed 3 times with PSB, incubated with DCFH-DA probe (probe: PBS=1:1000) for 30 minutes at room temperature, and then washed with PBS to excess untreated cells. bound probe. Finally, the fluorescence intensity of cells was monitored by flow cytometry to reflect the content of intracellular ROS.

### 2.12 Data statistics

Differences between the two groups were assessed using the Wilcoxon test, while correlation analysis was conducted using the Spearman correlation test. Survival analysis comparing the two groups was performed using Kaplan-Meier analysis and log-rank test. Cox regression analysis was performed using the R package “survival” to calculate hazard ratios (HRs) and 95% confidence intervals (CIs). All p-values were two-tailed, and a p-value less than 0.05 was considered statistically significant. Statistical analyses were conducted using R software (version 4.2.1).

## 3 Result

### 3.1 Screening key genes through WGCNA combined with differential analysis

**Figure 1:**
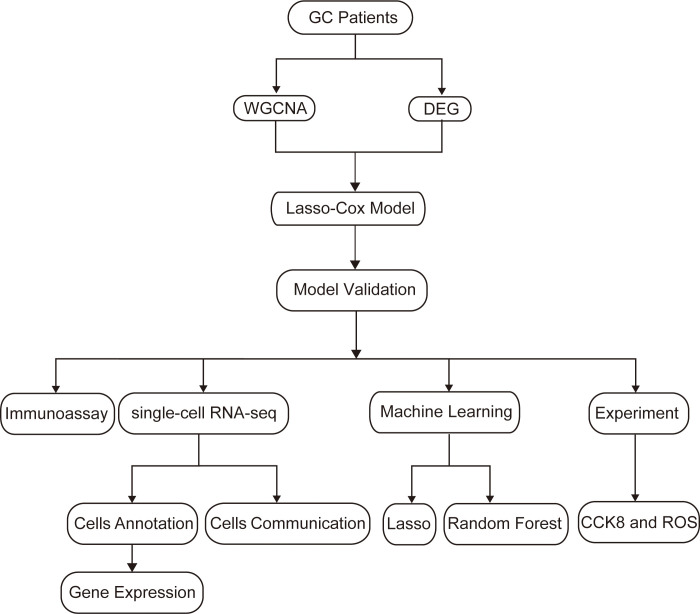
Flow chart.

**Figure 2:**
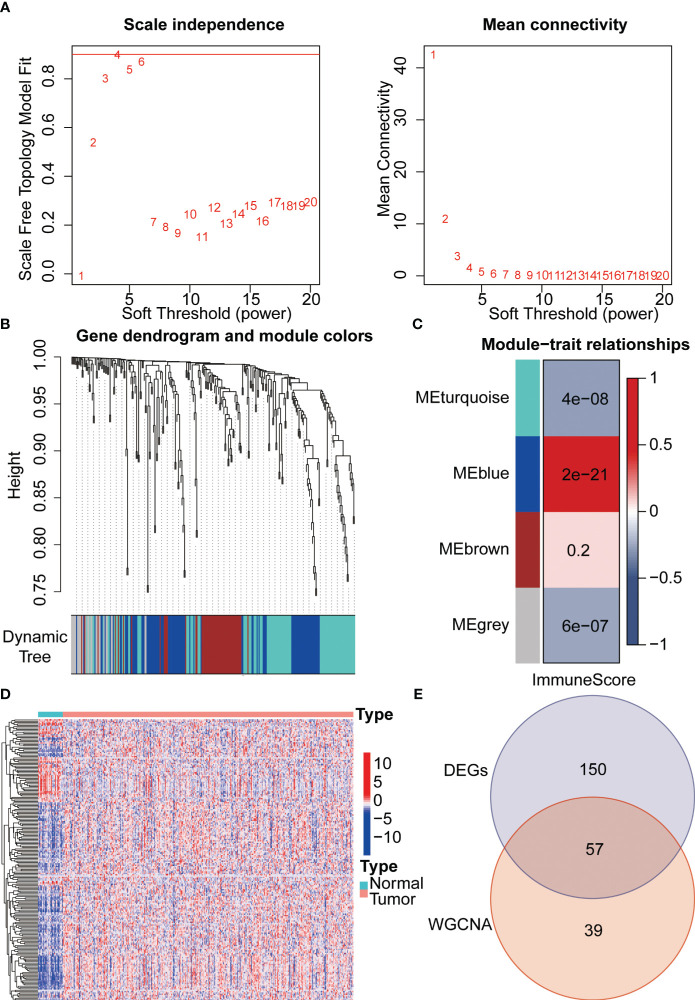
Integration of WGCNA and differential analysis for core gene selection. **(A)** Gene clustering was performed using the WGCNA algorithm. **(B)** Classification of all senescence-related genes into modules. **(C)** Identification of immune-related modules using the estimate algorithm. **(D)** Exploration of differential genes between gastric cancer and adjacent normal tissues through differential analysis. **(E)** Venn diagram illustrating the overlapping genes between WGCNA and differential analysis.

Firstly, we draw a flow chart to explain the whole analysis process (**Figure 1**). Considering the potential relevance of senescence-related genes to gastric cancer, we utilized these genes to construct a prognostic model. Firstly, we employed WGCNA to cluster the senescence-related genes, resulting in the identification of four modules (**Figure 2A**). Among these modules, MEblue module demonstrated a close association with tumor immunity, which was subsequently subjected to further analysis (**Figures 2B, C**). Subsequently, differential analysis was conducted to identify genes showing significant differences between cancer and adjacent tissues (**Figure 2D**). By taking the intersection between the genes in the MEblue module and the differentially expressed genes, a total of 57 genes were selected for subsequent analysis (**Figure 2E**).

### 3.2 Construction and validation of Lasso-Cox model

**Figure 3:**
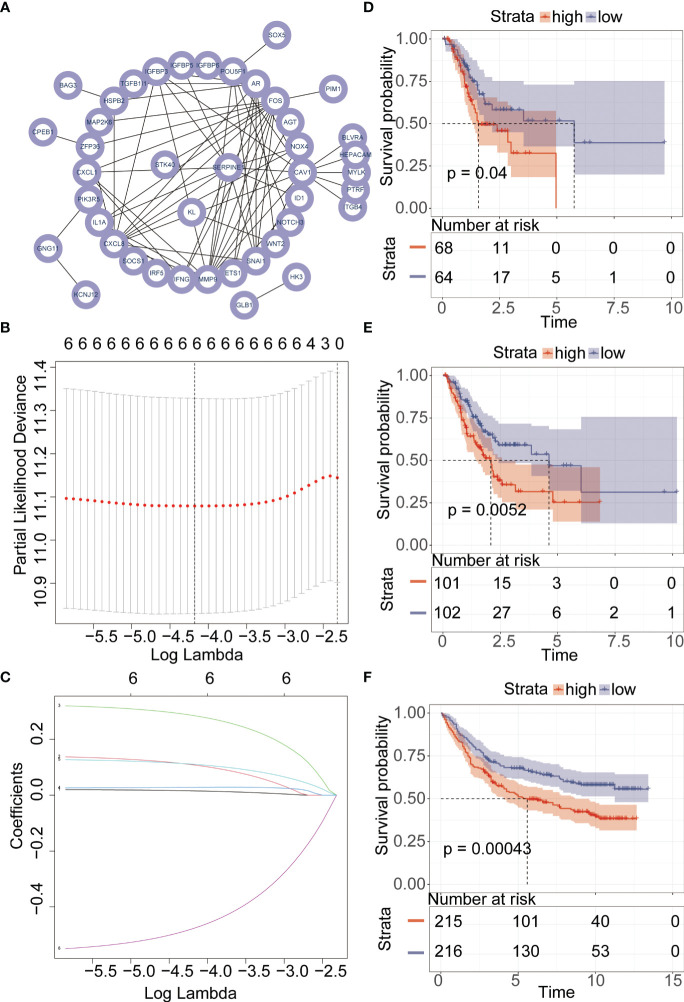
Lasso regression and Cox proportional hazards model. **(A)** Investigation of the association between genes within the MEblue module using the STRING database. **(B, C)** Key gene selection for model construction using the Lasso-Cox algorithm. **(D–F)** Kaplan-Meier analysis of the prognosis between high-risk and low-risk groups.

**Figure 4:**
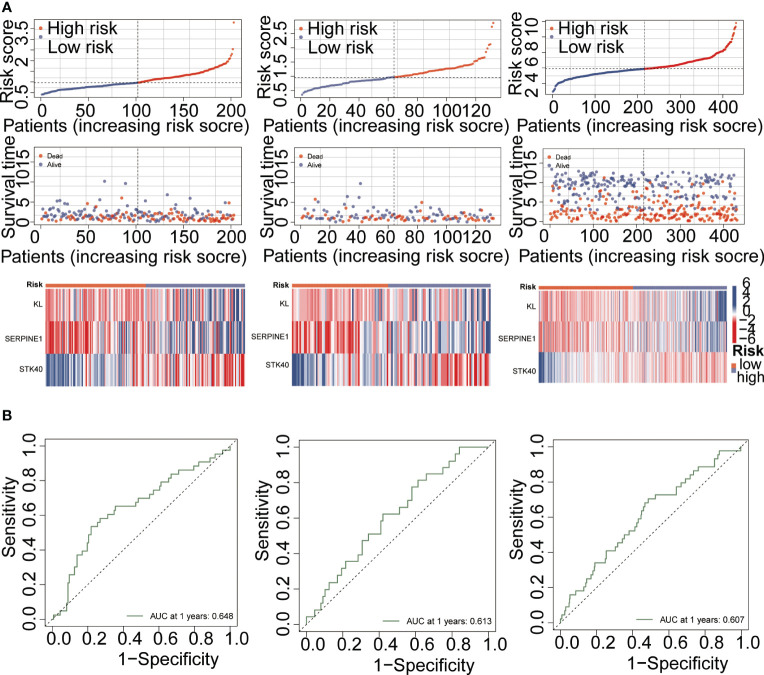
Model performance validation. **(A)** Risk curve demonstrating the differences between high-risk and low-risk groups. **(B)** ROC curve analysis evaluating the specific performance of the model.

**Figure 5:**
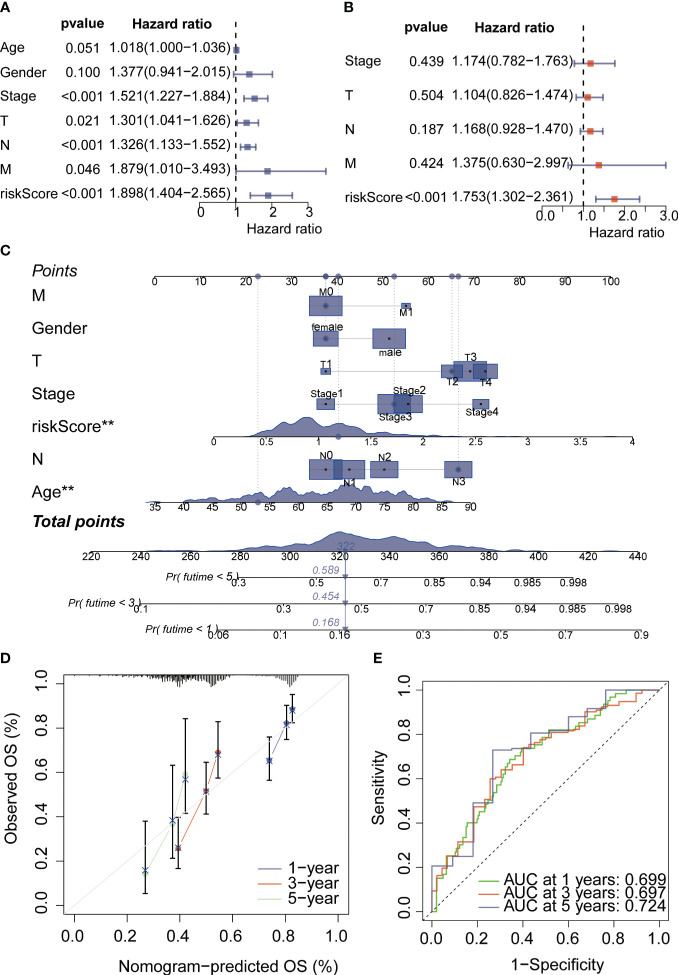
COX regression analysis and nomogram analysis. **(A)** Univariate COX regression analysis was conducted to evaluate valuable independent prognostic indicators. **(B)** Multivariate COX regression analysis was performed to assess valuable independent prognostic indicators. **(C)** The nomogram plot was used to estimate the probabilities of 1-year, 3-year, and 5-year survival rates for gastric cancer patients. **(D)** The calibration curve demonstrated satisfactory accuracy and predictive performance of the model. **(E)** ROC curve analysis was employed to evaluate the performance of the nomogram score assessment. *p < 0.05, **p < 0.01, ***p < 0.001, ****p < 0.0001. "ns" denotes not statistically significant.

PPI networks show the interactions between proteins (**Figure 3A**). We further investigated the interactions between different genes using the STRING database. Through Lasso-Cox regression analysis, we identified “KL,” “SERPINE1,” and “STK40” as key genes for model construction. The riskScore for each sample was calculated based on the formula (**Figures 3B, C**). Considering the correlation between this risk score and patient prognosis, we validated the model using three datasets. The TCGA dataset was divided into a training set and a validation set using the “caret” package, while the GSE84437 dataset served as an additional validation set. It can be observed that patients with high riskScore had poorer prognoses in all three datasets (**Figures 3D–F**). To further investigate the survival status of the two groups of patients, we found that the high-risk group had a higher mortality rate. Heatmap analysis revealed significantly higher expression levels of the three key genes, “KL,” “SERPINE1” and lower expression levels of “STK40” in the high-risk group compared to the low-risk group (**Figure 4A**). ROC curve analysis showed that the average AUC values for prognosis predictions on the TCGA training set reached 0.648, while the AUC values for the validation sets were 0.613 and 0.607 for the TCGA validation set and GSE84437 dataset, respectively (**Figure 4B**). Subsequently, we performed univariate and multivariate Cox regression analysis, and the results showed that riskScore was the only independent risk factor affecting the prognosis of gastric cancer patients (**Figures 5A, B**). In addition, we combined common clinical parameters and riskScore to construct a nomogram to predict the 1-year, 3-year, and 5-year survival rates of gastric cancer patients (**Figure 5C**). ROC curves and calibration curves indicated that the model had high accuracy and precision (**Figures 5D, E**).

### 3.3 Immunological landscape and drug sensitivity analysis of the model

**Figure 6:**
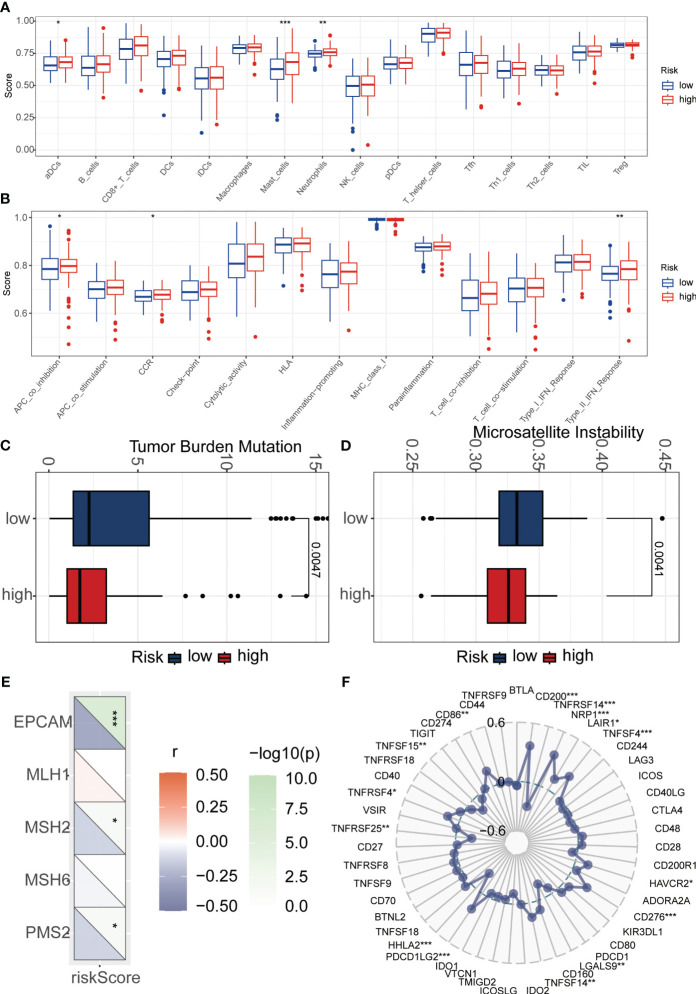
Immunological analysis between high and how-risk groups. **(A)** The ssGSEA algorithm was used to calculate the relationship between risk score and various immune cells. **(B)** The ssGSEA algorithm was employed to assess the relationship between risk score and different immune-related functions. **(C)** Tumor mutational burden (TMB) analysis was performed to evaluate the differences between high and low-risk groups. **(D)** Microsatellite instability (MSI) analysis was conducted to examine the differences between high and low-risk groups. **(E)** Mismatch repair (MMR) analysis revealed a strong association between risk score and MMR status. **(F)** Immune checkpoint analysis demonstrated a significant correlation between risk score and immune checkpoint expression. *p < 0.05, **p < 0.01, ***p < 0.001.

**Figure 7:**
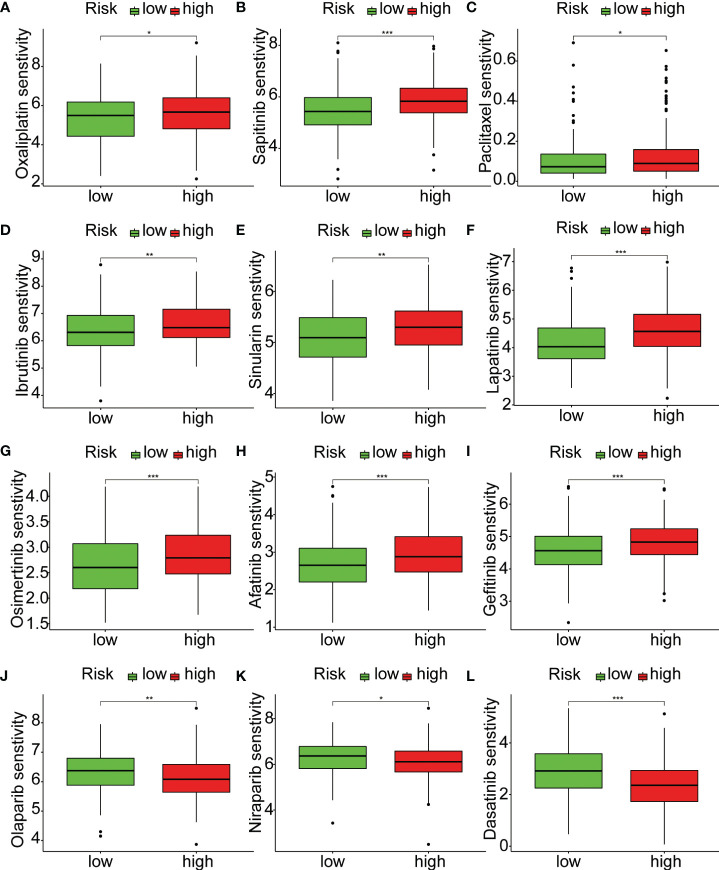
Relationship between riskScore and treatment sensitivity. **(A–L)** Analysis of differences in sensitivity to 12 drugs between high-risk and low-risk groups. *p < 0.05, **p < 0.01, ***p < 0.001.

To explore the relationship between riskScore and immune cells, we investigated the abundance of 16 immune cell types in the tumor microenvironment. The results showed that aDCs, Mast cells, and Neutrophils were significantly higher in the high-risk group compared to the low-risk group (**Figure 6A**). Next, we evaluated whether there were differences in immune-related functions between the high- and low-risk groups. We found that APC_co_inhibition, CCR, and Type_II_IFN_Response was significantly higher in the high-risk group compared to the low-risk group (**Figure 6B**). TMB refers to the number or frequency of mutations detected in tumor tissue. It is an indicator of the extent of genomic alterations in tumors, and high TMB is considered a potential predictor of immunotherapy response. We found that the high-risk group had lower TMB compared to the low-risk group (**Figure 6C**). High MSI is considered a potential predictor for immune checkpoint inhibitor therapy, such as PD-1 antibodies. Interestingly, our results showed that the low-risk group had higher MSI (**Figure 6D**). To understand the differences in immunotherapy between the high- and low-risk groups, we investigated whether the riskScore was associated with MMR and immune checkpoint markers. MMR analysis revealed a negative correlation between riskScore and EPCAM, MSH2, and PMS2 (**Figure 6E**). Immune checkpoint analysis also identified several markers that were significantly correlated with riskScore after adjusting the p-value to 0.001. HHLA2, PDCD1LG2, CD276, TNFSF4, NRP1, CD200, and TNFRSF14 remained closely associated with riskScore, while the commonly known PD1, PDL1, and CTLA4 did not show significant correlation (**Figure 6F**). Subsequently, we investigated the sensitivity of the high- and low-risk groups to different chemotherapy drugs. Interestingly, we found that Oxaliplatin, Sapitinib, Paclitaxel, Ibrutinib, Sinularin, Lapatinib, Osimertinib, Afatinib, and Gefitinib had higher IC50 values in the high-risk group (**Figures 7A–I**), while Olaparib, Niraparib, and Dasatinib had higher IC50 values in the low-risk group (**Figures 7J–L**).

### 3.4 Functional enrichment analysis

**Figure 8:**
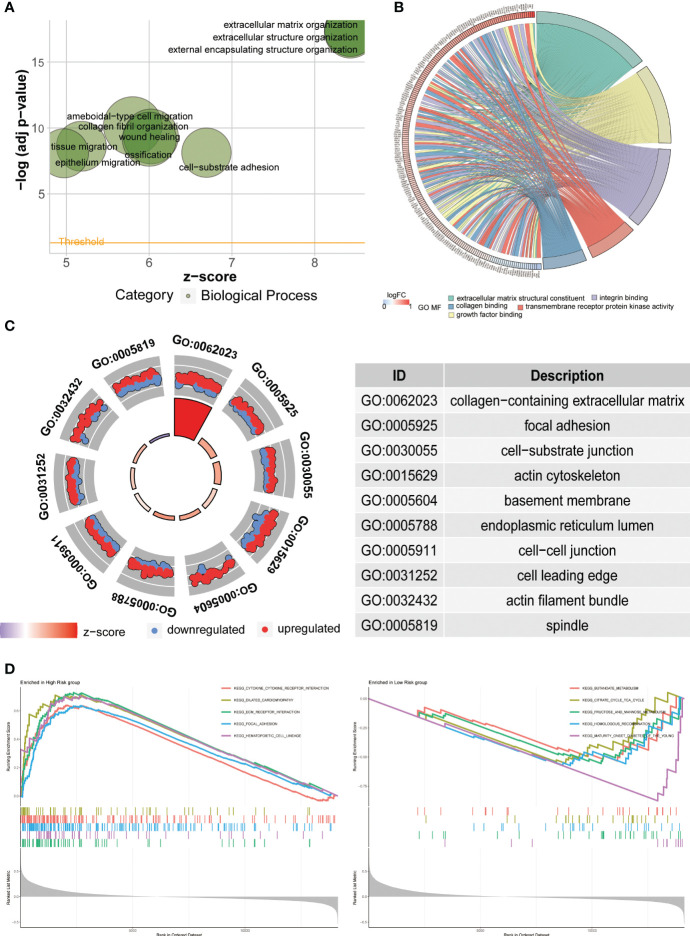
Functional enrichment analysis. **(A–C)** The results of Gene Ontology (GO) analysis illustrate the enriched functions in Biological Processes (BP), Molecular Functions (MF), and Cellular Components (CC). **(D)** The Gene Set Enrichment Analysis (GSEA) is employed to explore potential mechanisms and pathways based on the Kyoto Encyclopedia of Genes and Genomes (KEGG) database.

Functional enrichment analysis, such as Gene Ontology (GO) analysis, allows us to further investigate the functional characteristics of disease-related genes, differentially expressed genes under specific conditions, and important functional modules in gene regulatory networks. In the Biological Process (BP) analysis, we observed enrichment in extracellular structure organization (**Figure 8A**). The Molecular Function (MF) analysis showed enrichment in extracellular matrix structural constituent and growth factor binding (**Figure 8B**). The Cellular Component (CC) analysis revealed enrichment in collagen-containing extracellular matrix and cell-substrate junction (**Figure 8C**). Furthermore, we performed Gene Set Enrichment Analysis (GSEA) to further explore the pathways enriched in the high- and low-risk groups. The high-risk group was enriched in pathways such as CYTOKINE_CYTOKINE_RECEPTOR_INTERACTION, ECM_RECEPTORINTERACTION, and FOCAL_ADHESION, while the low-risk group was mainly enriched in pathways such as KEGG_BUTANOATE_METABOLISM and KEGG_CITRATE_CYCLE_TCA_CYCLE (**Figure 8D**).

### 3.5 Single-cell data analysis

**Figure 9:**
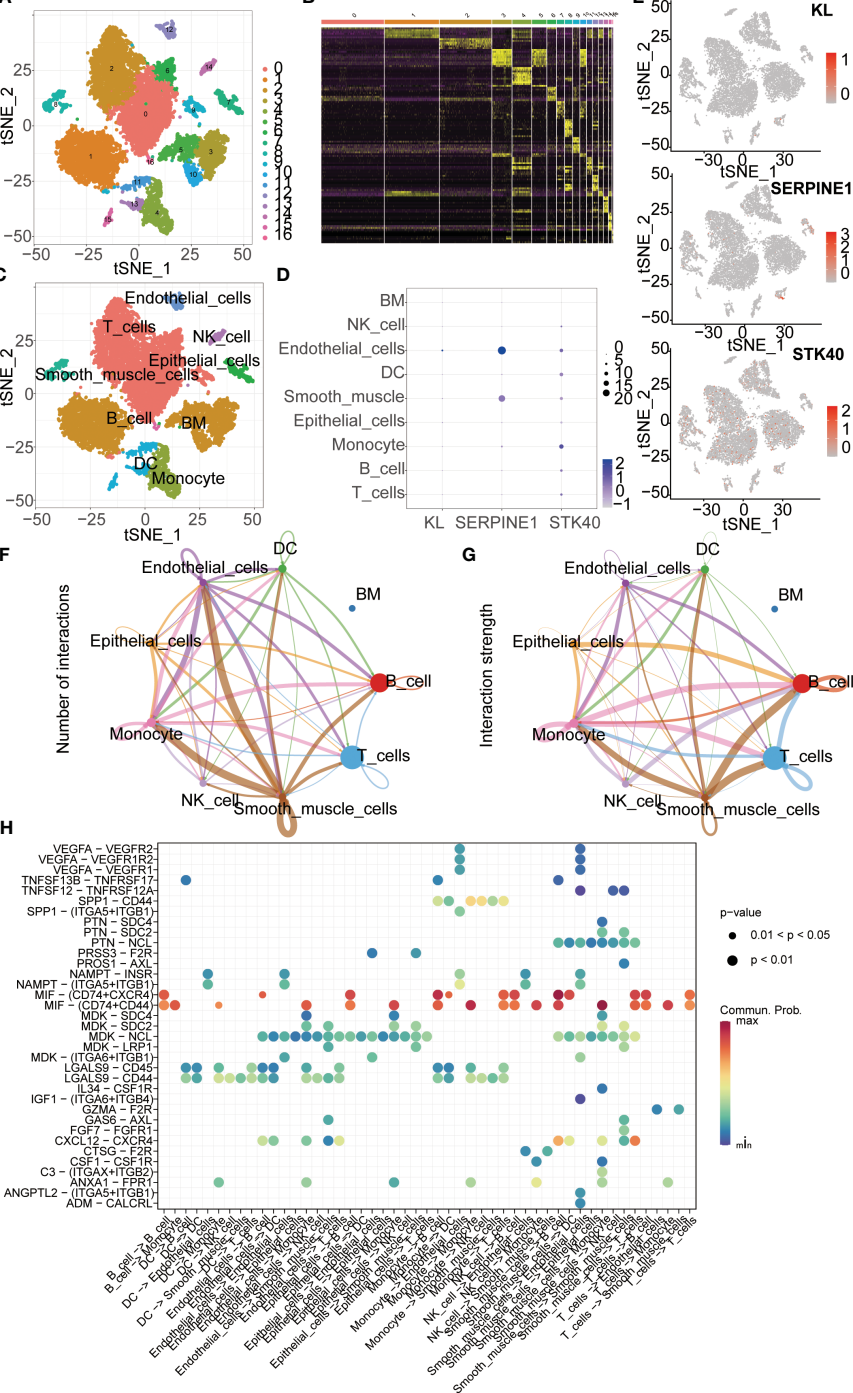
ScRNA-seq Data Analysis. **(A)** t-SNE clustering algorithm is used to classify cells into 17 clusters. **(B)** Heatmap visualizes the differentially expressed genes among the identified clusters. **(C)** “SingleR” package is employed to annotate different cell types, including T cells, B cells, Monocytes, Epithelial cells, Smooth muscle cells, Dendritic cells, Endothelial cells, NK cells, and Bone marrow cells. **(D, E)** ScRNA-seq analysis reveals the expression patterns of key genes across different cell types. **(F)** The “cellchat” package is utilized to investigate the number of interactions between different cell types. **(G)** The “cellchat” package is employed to study the strength of interactions between different cell types. **(H)** The analysis focuses on exploring the molecular mechanisms underlying the interactions between different cell types.

**Figure 10:**
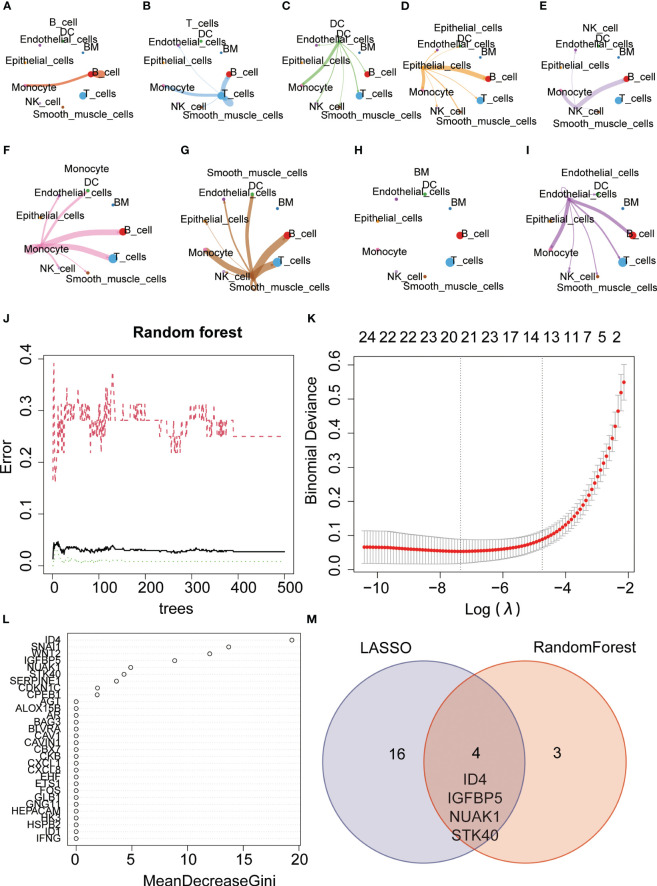
Gene selection using machine learning algorithms. **(A–I)** Communication network diagram between different cells **(J, K)** Key genes were selected using the random forest algorithm. **(L)** Key genes were selected using the Lasso algorithm. **(M)** The VENN diagram illustrates the intersection of genes identified by both algorithms, including ID4, IGFBP5, NUAK1, and STK40.

We downloaded scRNA-seq data of gastric cancer tissue from the GEO database (GSE167297). After data processing, we obtained a total of 10 gastric cancer samples for further analysis. PCA and tSNE dimensionality reduction analyses were performed, resulting in the identification of 17 clusters among the samples. Subsequent heatmap analysis revealed differential gene expression patterns between these clusters (**Figures 9A, B**). Next, we annotated the 17 cell clusters and classified them into 9 main cell types, including T cells, B cells, Monocytes, Epithelial cells, Smooth muscle cells, Dendritic cells (DCs), Endothelial cells, NK cells, and Bone Marrow (BM) cells (**Figure 9C**). We then examined the expression levels of key genes used for modeling across different cell types in gastric cancer tissue. Scatter plots clearly showed that KL had the highest expression in Endothelial cells, SERPINE1 was mainly expressed in Endothelial cells and Smooth muscle cells, and STK40 was predominantly expressed in Monocytes (**Figures 9D, E**). Cellular communication is a fundamental process in which cells within an organism interact and coordinate through the transmission of molecular signals. It plays a paramount role in the organism, serving as a crucial mechanism for intercellular coordination and regulation. Furthermore, we investigated the intercellular communication between different cell types. The interactions between Smooth muscle cells and Monocytes, as well as between Smooth muscle cells and Endothelial cells, were the most abundant. Additionally, the interaction strength between Smooth muscle cells and B cells was the strongest (**Figures 9F, G**, **10A–I**). Subsequently, we explored the molecular mediators of intercellular interactions. The interaction between Smooth muscle cells and B cells was primarily mediated through the MIF signaling pathway involving CD74 and CXCR4. The interaction between Smooth muscle cells and Monocytes was mediated through the MIF signaling pathway involving CD74 and CD44 (**Figure 9H**).

### 3.6 Machine learning-based hub gene selection

The Random Forest is an ensemble learning approach that enhances predictive accuracy and stability by amalgamating the predictive outcomes of individual decision trees. We further employed the random forest machine learning algorithm to screen for key genes and identified a set of 7 genes (**Figures 10J, K**). Additionally, the Lasso regression algorithm identified 20 key genes (**Figure 10L**). Taking the intersection of the two algorithms, we obtained four common genes, namely ID4, IGFBP5, NUAK1, and STK40. Interestingly, STK40 was also one of the three key genes identified in the previous modeling process (**Figure 10M**). Therefore, we believe that STK40 is one of the most critical senescence-related genes in gastric cancer, which needs further study.

### 3.7 STK40 expression verification and *in vitro* function exploration

**Figure 11:**
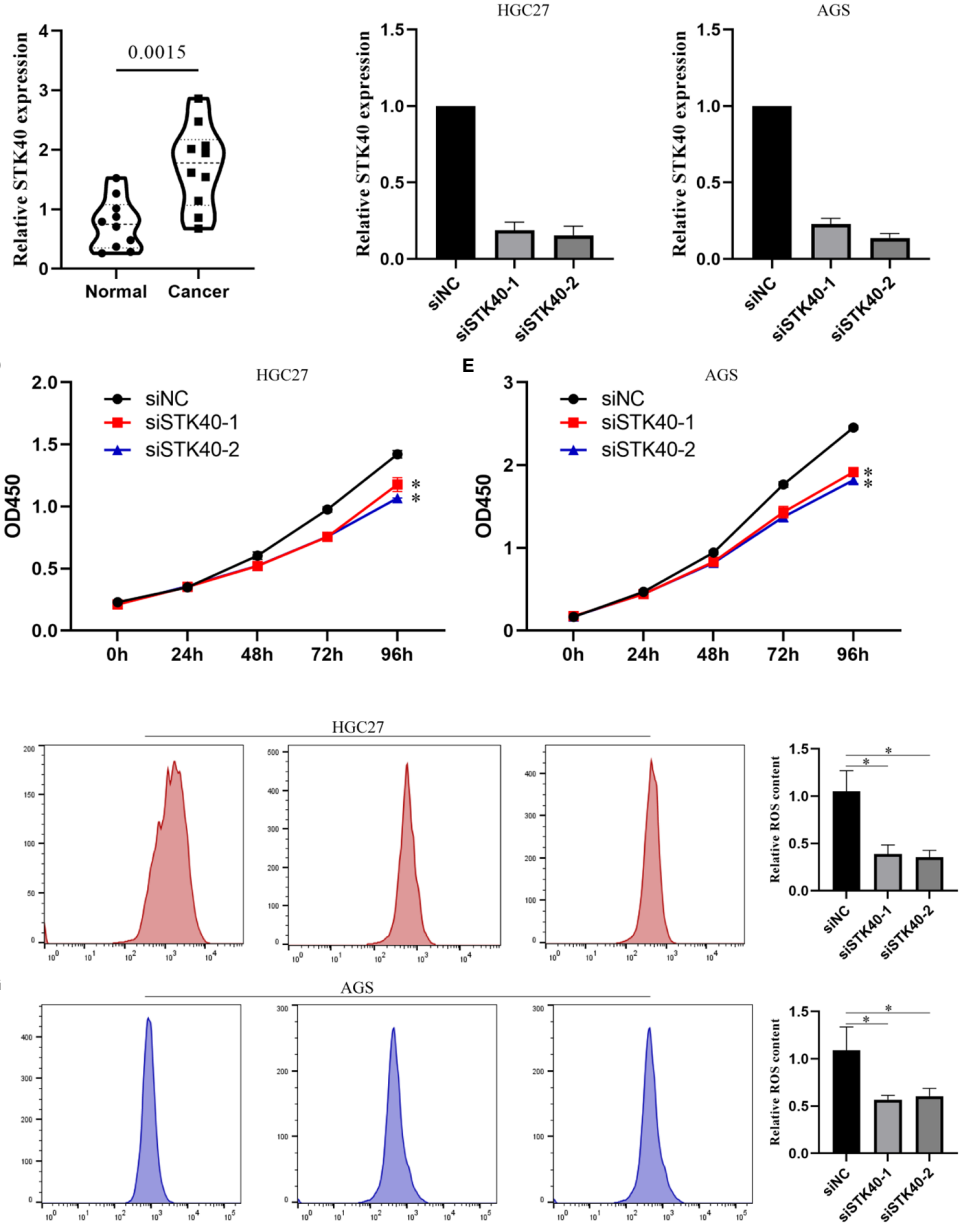
Biological function of STK40 in CRC. **(A)** Differential expression of STK40 in CRC. **(B, C)** Knockdown efficiency of siSTK40-1 and siSTK40-2 in gastric cancer cells. **(D, E)** The proliferation ability of gastric cancer cell lines is impaired after STK40 knockdown. **(F, G)** Reduced ROS accumulation in gastric cancer cell lines after STK40 knockdown. *p < 0.05, **p < 0.01.

First, we explored the expression of STK40 in 10 pairs of gastric cancer tissues, and the results showed that the expression of STK40 in gastric cancer tissues was significantly higher than that in adjacent normal tissues (**Figure 11A**). Then, we deeply explored the biological function of STK40 in gastric cancer. When we knocked down STK40 expression in gastric cancer cells using siSTK40-1 and siSTK40-2, the proliferation ability of gastric cancer cells was significantly impaired (**Figures 11B–E**). More importantly, we also detected the effect of reduced expression of STK40 on ROS content. The results showed that the accumulation of ROS in gastric cancer cells was significantly impaired after the expression of STK40 was reduced (**Figures 11F, G**).

## 4 Discussion

Gastric cancer is a common and highly lethal malignant tumor that primarily originates from the epithelial cells of the gastric mucosa (24). Due to its diverse subtypes and molecular subgroups, the diagnosis and treatment of gastric cancer pose significant challenges (5). Immunotherapy has emerged as a revolutionary cancer treatment modality, demonstrating promising efficacy in a subset of patients. However, numerous studies have indicated that immunotherapy is effective only in a proportion of individuals (12, 13). In order to gain further insights into the prognosis of different gastric cancer patients and the efficacy of immunotherapy, we constructed a prognostic model using hub genes and utilized it to predict the therapeutic response to immunotherapy.

Considering the potential association between senescence-related genes and gastric cancer, we employed the WGCNA algorithm to identify immune-related senescence gene modules. Subsequently, a Lasso-Cox model was constructed using the senescence-related genes within this module, including KL, SERPINE1, and STK40. Our findings revealed that downregulation of KL gene expression promotes cell proliferation and contributes to the progression of gastric cancer (25). Furthermore, SERPINE1 is typically upregulated in gastric cancer tissues and its overexpression is associated with increased tumor invasiveness and poor prognosis (26). High expression of STK40 has been closely correlated with the occurrence and development of esophageal cancer (27). These research findings provide further validation for the reliability of our selected genes and are consistent with our prediction results. Subsequently, we performed survival analysis of the model using three independent datasets. The results revealed a significantly poorer prognosis for patients in the high-risk group compared to the low-risk group. Furthermore, the predictive performance of the model was assessed using ROC curve analysis, which demonstrated good accuracy. Moreover, additional COX regression analysis confirmed that the risk score derived from the model was an independent prognostic factor of significance.

Using the ssGSEA algorithm, we investigated the relationship between the risk score of our model and 16 immune cell types and 13 immune-related functions. We observed that aDCs, Mast cells, and Neutrophils exhibited significantly higher expression levels in the high-risk group compared to the low-risk group. Previous studies have shown that under the stimulation of IL33, activated Mast cells can activate macrophages, thereby promoting the development of gastric cancer (28). Additionally, Wang et al. found a significant increase in the infiltration of Neutrophils in tumor tissues of gastric cancer patients (29). Immunotherapy has emerged as a revolutionary approach for cancer treatment; however, its efficacy varies among different solid tumor types. Therefore, identifying biomarkers that can predict patient response to immunotherapy is of utmost importance (13). TMB and microsatellite MSI are commonly used indicators for predicting immunotherapy response (30, 31). In our study, we found that the high-risk group had lower TMB and MSI levels compared to the low-risk group. Previous studies have indicated that high TMB and MSI are associated with increased mutational load and neoantigen production, suggesting that the high-risk group may have a reduced response to immunotherapy. MMR status is another commonly used predictor of immunotherapy response (32). We observed a strong correlation between risk score and MMR-related markers, including EPCAM, MSH2, and PMS2. Among these markers, EPCAM showed the highest correlation with the risk score. Previous studies have also demonstrated a close association between EPCAM and the prognosis of gastric cancer (33). Furthermore, we assessed the relationship between risk score and immune checkpoints. The results revealed a close correlation between risk score and immune checkpoints such as HHLA2, PDCD1LG2, CD276, TNFSF4, NRP1, CD200, and TNFRSF14. However, commonly studied immune checkpoints like PD1, PDL1, and CTLA4 did not exhibit significant correlations. This suggests the importance of considering other immune checkpoints beyond PD1, PDL1, and CTLA4 when making clinical treatment decisions.

To investigate the differences between the high-risk and low-risk groups, we conducted GO and KEGG analyses to explore potential mechanistic pathways. The GO analysis primarily revealed enrichment in extracellular matrix organization, highlighting the crucial role of the extracellular matrix (ECM) in the initiation and progression of gastric cancer (34, 35). Notably, the KEGG analysis identified several enriched pathways, including CYTOKINE_CYTOKINE_RECEPTOR_INTERACTION, ECM_RECEPTOR_INTERACTION, FOCAL_ADHESION, KEGG_BUTANOATE_METABOLISM, and KEGG_CITRATE_CYCLE_TCA_CYCLE. Remarkably, these findings align closely with our GO analysis, further emphasizing the significant involvement of the ECM. Specifically, these pathways underscore the importance of cytokine signaling, ECM-receptor interaction, focal adhesion, as well as metabolic processes like butanoate metabolism and the citrate cycle (TCA cycle) in GC development. Taken together, our findings provide compelling evidence for the pivotal role of the ECM in GC and support the notion that ECM-related processes contribute significantly to the molecular landscape and potential therapeutic targets in GC.

Single-cell sequencing is a high-resolution genomics technique that enables the characterization of cellular heterogeneity and differences across different cell types (36). In order to investigate the expression patterns of key molecules used in model construction across different cell types, we conducted single-cell analysis using the GSE167297 dataset. We observed relatively low expression levels of the KL gene in various cell types, with the highest expression observed in Endothelial cells. On the other hand, SERPINE1 was mainly expressed in Endothelial cells and Smooth muscle cells, while STK40 showed predominant expression in Monocytes. Previous studies have indicated a close association between the proportion of Monocytes and the prognosis of gastric cancer (37). Cellular communication plays a crucial role in mediating molecular signaling and regulation between cells, and is essential for maintaining normal cellular functions, tissue development, and homeostasis (38, 39). We analyzed the communication between different cell types in gastric cancer tissues and explored potential interacting molecules. This provided valuable insights for further exploration of the underlying mechanisms. Subsequently, we employed two machine learning algorithms, namely random forest and lasso, to identify key genes associated with senescence. By intersecting the results from both algorithms, we identified four core genes, namely ID4, IGFBP5, NUAK1, and STK40. It is worth noting that STK40, as a key gene in model construction, still lacks relevant research in gastric cancer. Therefore, we further explored whether STK40 acts as one of the factors affecting gastric cancer progression. The results showed that the proliferation ability of gastric cancer cell lines was significantly weakened after the expression of STK40 was reduced. ROS often play a double-edged sword role in cancer, and whether and how STK40 affects ROS accumulation was previously unknown. Therefore, we further explored whether STK40 affects intracellular ROS content, and the results showed that reduced expression of STK40 could significantly reduce ROS accumulation in gastric cancer cells. This provides a partial reference for the mechanism by which STK40 affects the proliferation ability of gastric cancer cells.

Certainly, this study carries significant clinical implications as it utilizes a comprehensive approach combining bulk sequencing and scRNA-seq analyses. The risk score derived from the integration of WGCNA and Lasso-Cox algorithms emerges as a robust and independent biomarker with prognostic value for gastric cancer patients. Moreover, the ability to evaluate differential drug sensitivity between high- and low-risk groups based on the risk model holds promise for tailoring personalized chemotherapy regimens. Furthermore, the utilization of scRNA-seq analysis allows for a more detailed exploration of the expression patterns of hub genes across distinct cell types. This comprehensive understanding enhances our insights into the intricate cellular landscape of gastric cancer. Additionally, through functional enrichment analysis, we gain deeper insights into the underlying molecular mechanisms and downstream pathways associated with the risk model, providing a foundation for further mechanistic investigations.

In conclusion, we have developed a model based on key genes that can predict the prognosis of gastric cancer. Furthermore, this model demonstrates effectiveness in predicting the immunotherapy response of patients.

## Data availability statement

The datasets presented in this study can be found in online repositories. The names of the repository/repositories and accession number(s) can be found in the article/**Supplementary Material**.

## Ethics statement

The studies involving humans were approved by The Ethics Committee of Tongren Hospital, Shanghai Jiao Tong University School of Medicine. The studies were conducted in accordance with the local legislation and institutional requirements. The participants provided their written informed consent to participate in this study.

## Author contributions

WS: Data curation, Methodology, Validation, Writing – original draft. YY: Validation, Writing – original draft, Data curation. JC: Visualization, Writing – original draft. QB: Visualization, Writing – original draft. MS: Data curation, Writing – original draft. PS: Conceptualization, Funding acquisition, Resources, Writing – review & editing. HP: Conceptualization, Funding acquisition, Resources, Supervision, Writing – review & editing.

## References

[1] Ajani JA D'Amico TA Bentrem DJ Chao J Cooke D Corvera C. Gastric cancer, version 2.2022, NCCN clinical practice guidelines in oncology. J Natl Compr Canc Netw (2022) 20(2):167–92. doi: 10.6004/jnccn.2022.0008 35130500

[2] Smyth EC Nilsson M Grabsch HI van Grieken NC Lordick F. Gastric cancer. Lancet (2020) 396(10251):635–48. doi: 10.1016/S0140-6736(20)31288-5 32861308

[3] Rocken C. Predictive biomarkers in gastric cancer. J Cancer Res Clin Oncol (2023) 149(1):467–81. doi: 10.1007/s00432-022-04408-0 PMC9889517 36260159

[4] Lordick F Carneiro F Cascinu S Fleitas T Haustermans K Piessen G. Gastric cancer: ESMO Clinical Practice Guideline for diagnosis, treatment and follow-up. Ann Oncol (2022) 33(10):1005–20. doi: 10.1016/j.annonc.2022.07.004 35914639

[5] Zeng Y Jin RU. Molecular pathogenesis, targeted therapies, and future perspectives for gastric cancer. Semin Cancer Biol (2022) 86(Pt 3):566–82. doi: 10.1016/j.semcancer.2021.12.004 PMC12833737 34933124

[6] Chia NY Tan P. Molecular classification of gastric cancer. Ann Oncol (2016) 27(5):763–9. doi: 10.1093/annonc/mdw040 26861606

[7] Onoyama T Ishikawa S Isomoto H. Gastric cancer and genomics: review of literature. J Gastroenterol (2022) 57(8):505–16. doi: 10.1007/s00535-022-01879-3 PMC9308599 35751736

[8] Chow A Perica K Klebanoff CA Wolchok JD. Clinical implications of T cell exhaustion for cancer immunotherapy. Nat Rev Clin Oncol (2022) 19(12):775–90. doi: 10.1038/s41571-022-00689-z PMC10984554 36216928

[9] Zhu S Zhang T Zheng L Liu H Song W Liu D. Combination strategies to maximize the benefits of cancer immunotherapy. J Hematol Oncol (2021) 14(1):156. doi: 10.1186/s13045-021-01164-5 34579759 PMC8475356

[10] Lesch S Gill S. The promise and perils of immunotherapy. Blood Adv (2021) 5(18):3709–25. doi: 10.1182/bloodadvances.2021004453C PMC8945582 34581774

[11] Markl F Huynh D Endres S Kobold S. Utilizing chemokines in cancer immunotherapy. Trends Cancer. (2022) 8(8):670–82. doi: 10.1016/j.trecan.2022.04.001 35501268

[12] Bagchi S Yuan R Engleman EG. Immune checkpoint inhibitors for the treatment of cancer: clinical impact and mechanisms of response and resistance. Annu Rev Pathol (2021) 16:223–49. doi: 10.1146/annurev-pathol-042020-042741 33197221

[13] Jin X Liu Z Yang D Yin K Chang X. Recent progress and future perspectives of immunotherapy in advanced gastric cancer. Front Immunol (2022) 13:948647. doi: 10.3389/fimmu.2022.948647 35844558 PMC9284215

[14] Bejarano L Jordao MJC Joyce JA. Therapeutic targeting of the tumor microenvironment. Cancer Discovery (2021) 11(4):933–59. doi: 10.1158/2159-8290.CD-20-1808 33811125

[15] Xiao Y Yu D. Tumor microenvironment as a therapeutic target in cancer. Pharmacol Ther (2021) 221:107753. doi: 10.1016/j.pharmthera.2020.107753 33259885 PMC8084948

[16] Campisi J. Aging, cellular senescence, and cancer. Annu Rev Physiol (2013) 75:685–705. doi: 10.1146/annurev-physiol-030212-183653 23140366 PMC4166529

[17] Calcinotto A Kohli J Zagato E Pellegrini L Demaria M Alimonti A. Cellular senescence: aging, cancer, and injury. Physiol Rev (2019) 99(2):1047–78. doi: 10.1152/physrev.00020.2018 30648461

[18] Huang W Hickson LJ Eirin A Kirkland JL Lerman LO. Cellular senescence: the good, the bad and the unknown. Nat Rev Nephrol. (2022) 18(10):611–27. doi: 10.1038/s41581-022-00601-z PMC9362342 35922662

[19] Zhang H Zhang X Li X Meng WB Bai ZT Rui SZ. Effect of CCNB1 silencing on cell cycle, senescence, and apoptosis through the p53 signaling pathway in pancreatic cancer. J Cell Physiol (2018) 234(1):619–31. doi: 10.1002/jcp.26816. 30069972

[20] Sun JX Liu CQ Xu JZ An Y Xu MY Zhong XY. A four-cell-senescence-regulator-gene prognostic index verified by genome-wide CRISPR can depict the tumor microenvironment and guide clinical treatment of bladder cancer. Front Immunol (2022) 13:908068. doi: 10.3389/fimmu.2022.908068 35898492 PMC9312376

[21] Liu B Zhou Z Jin Y Lu J Feng D Peng R. Hepatic stellate cell activation and senescence induced by intrahepatic microbiota disturbances drive progression of liver cirrhosis toward hepatocellular carcinoma. J Immunother Cancer (2022) 10(1):e003069. doi: 10.1136/jitc-2021-003069 PMC8744134 34996812

[22] Yu L Cao C Li X Zhang M Gu Q Gao H. Complete loss of miR-200 family induces EMT associated cellular senescence in gastric cancer. Oncogene (2022) 41(1):26–36. doi: 10.1038/s41388-021-02067-y 34667277 PMC8724006

[23] Sun W Wang J Wang Z Xu M Lin Q Sun P. Combining WGCNA and machine learning to construct basement membrane-related gene index helps to predict the prognosis and tumor microenvironment of HCC patients and verifies the carcinogenesis of key gene CTSA. Front Immunol (2023) 14:1185916. doi: 10.3389/fimmu.2023.1185916 37287981 PMC10242074

[24] Norwood DA Montalvan-Sanchez E Dominguez RL Morgan DR. Gastric cancer: emerging trends in prevention, diagnosis, and treatment. Gastroenterol Clin North Am (2022) 51(3):501–18. doi: 10.1016/j.gtc.2022.05.001 36153107

[25] Wang L Wang X Wang X Jie P Lu H Zhang S. Klotho is silenced through promoter hypermethylation in gastric cancer. Am J Cancer Res (2011) 1(1):111–9. PMC3180103 21969138

[26] Yang JD Ma L Zhu Z. SERPINE1 as a cancer-promoting gene in gastric adenocarcinoma: facilitates tumour cell proliferation, migration, and invasion by regulating EMT. J Chemother (2019) 31(7-8):408–18. doi: 10.1080/1120009X.2019.1687996 31724495

[27] Fong LY Huebner K Jing R Smalley KJ Brydges CR Fiehn O. Zinc treatment reverses and anti-Zn-regulated miRs suppress esophageal carcinomas *in vivo* . Proc Natl Acad Sci U.S.A. (2023) 120(20):e2220334120. doi: 10.1073/pnas.2220334120 37155893 PMC10193985

[28] Eissmann MF Dijkstra C Jarnicki A Phesse T Brunnberg J Poh AR. IL-33-mediated mast cell activation promotes gastric cancer through macrophage mobilization. Nat Commun (2019) 10(1):2735. doi: 10.1038/s41467-019-10676-1 31227713 PMC6588585

[29] Wang TT Zhao YL Peng LS Chen N Chen W Lv YP. Tumour-activated neutrophils in gastric cancer foster immune suppression and disease progression through GM-CSF-PD-L1 pathway. Gut (2017) 66(11):1900–11. doi: 10.1136/gutjnl-2016-313075 PMC5739867 28274999

[30] Chalmers ZR Connelly CF Fabrizio D Gay L Ali SM Ennis R. Analysis of 100,000 human cancer genomes reveals the landscape of tumor mutational burden. Genome Med (2017) 9(1):34. doi: 10.1186/s13073-017-0424-2 28420421 PMC5395719

[31] Hause RJ Pritchard CC Shendure J Salipante SJ. Classification and characterization of microsatellite instability across 18 cancer types. Nat Med (2016) 22(11):1342–50. doi: 10.1038/nm.4191 27694933

[32] He Y Zhang L Zhou R Wang Y Chen H. The role of DNA mismatch repair in immunotherapy of human cancer. Int J Biol Sci (2022) 18(7):2821–32. doi: 10.7150/ijbs.71714 PMC9066103 35541922

[33] Dai M Yuan F Fu C Shen G Hu S Shen G. Relationship between epithelial cell adhesion molecule (EpCAM) overexpression and gastric cancer patients: A systematic review and meta-analysis. PloS One (2017) 12(4):e0175357. doi: 10.1371/journal.pone.0175357 28403178 PMC5389808

[34] Wang X Shi X Lu H Zhang C Li X Zhang T. Succinylation inhibits the enzymatic hydrolysis of the extracellular matrix protein fibrillin 1 and promotes gastric cancer progression. Adv Sci (Weinh). (2022) 9(27):e2200546. doi: 10.1002/advs.202200546 35901491 PMC9507347

[35] Zhang T Li X He Y Wang Y Shen J Wang S. Cancer-associated fibroblasts-derived HAPLN1 promotes tumour invasion through extracellular matrix remodeling in gastric cancer. Gastric Cancer. (2022) 25(2):346–59. doi: 10.1007/s10120-021-01259-5 PMC8882084 34724589

[36] Regner MJ Wisniewska K Garcia-Recio S Thennavan A Mendez-Giraldez R Malladi VS. A multi-omic single-cell landscape of human gynecologic Malignancies. Mol Cell (2021) 81(23):4924–41 e10. doi: 10.1016/j.molcel.2021.10.013 34739872 PMC8642316

[37] Ma JY Liu Q. Clinicopathological and prognostic significance of lymphocyte to monocyte ratio in patients with gastric cancer: A meta-analysis. Int J Surg (2018) 50:67–71. doi: 10.1016/j.ijsu.2018.01.002 29329786

[38] Jin S Guerrero-Juarez CF Zhang L Chang I Ramos R Kuan CH. Inference and analysis of cell-cell communication using CellChat. Nat Commun (2021) 12(1):1088. doi: 10.1038/s41467-021-21246-9 33597522 PMC7889871

[39] Fang Z Tian Y Sui C Guo Y Hu X Lai Y. Single-cell transcriptomics of proliferative phase endometrium: systems analysis of cell-cell communication network using cellchat. Front Cell Dev Biol (2022) 10:919731. doi: 10.3389/fcell.2022.919731 35938159 PMC9352955

